# Incidence and risk factors of acute skin failure in patients with sepsis: a systematic review and meta-analysis

**DOI:** 10.3389/fpubh.2026.1955939

**Published:** 2026-10-14

**Authors:** Wenshuang Wang, Tian Lang, Kai Zhao, Hong Zhang, Huan Shi, Yuan Yuan, Wei Cao

**Affiliations:** 1 Department of Nursing, Beijing TianTan Hospital, Capital Medical University, Beijing, China; 2 Department of Intensive Care Unit, Beijing TianTan Hospital, Capital Medical University, Beijing, China

**Keywords:** acute skin failure, critical care, incidence, meta-analysis, risk factor, sepsis

## Abstract

**Purpose:**

This systematic review and meta-analysis aimed to determine the incidence and risk factors of acute skin failure (ASF) in patients with sepsis.

**Methods:**

Two reviewers independently searched PubMed, Web of Science, Cochrane Database, Embase, CNKI, Wanfang Database, and VIP Database from database inception to December 2025. Observational studies reporting ASF incidence and risk factors in sepsis patients were included. Random-effects meta-analysis was used to pool incidence and odds ratios (ORs); case–control studies were excluded from incidence pooling and analyzed only for associated factors. Publication bias was assessed using funnel plots, Begg’s and Egger’s tests. Study quality was evaluated using the Newcastle-Ottawa Scale (NOS) and the AHRQ checklist.

**Results:**

Eighteen observational studies comprising 3,295 adult participants were included. After exclusion of two case–control studies from incidence pooling, the pooled ASF incidence across the 16 cohort and cross-sectional studies (*n* = 2,697) was 45.2% (95% CI: 34.5–56.2%), with a 95% prediction interval of 12.2–83.0% and substantial heterogeneity (*I*^2^ = 96.1%, *p* < 0.0001). Elevated arterial blood lactate [OR 1.20, 95% CI 1.15–1.25; *p* < 0.00001; *I*^2^ = 50%], higher SOFA score [OR 1.23, 95% CI 1.16–1.31; *p* < 0.00001; *I*^2^ = 20%], and higher APACHE II score [OR 1.16, 95% CI 1.10–1.21; *p* < 0.00001; *I*^2^ = 34%] were significantly associated with increased ASF risk, whereas lower mean arterial pressure was associated with ASF [OR 0.94, 95% CI 0.93–0.96; *p* < 0.00001; *I*^2^ = 47%]. Leave-one-out sensitivity analyses confirmed the robustness of the pooled estimates, and no significant publication bias was detected (Egger’s *p* = 0.467; Begg’s *p* = 0.239).

**Conclusion:**

ASF affects a substantial proportion of patients with sepsis. Lactate, SOFA score, APACHE II score and MAP are variables associated with ASF that may support early identification of patients at heightened risk; prospective studies are needed to validate predictive models.

## 1 Introduction

Sepsis is a life-threatening condition arising from a dysregulated host response to infection (1) and affects approximately 48.9 million individuals worldwide each year, causing about 11 million deaths (2). Systemic vasodilation, capillary leak, coagulation activation, and microcirculatory dysfunction impair cellular oxygen delivery (3, 4); the resulting tissue hypoperfusion is clinically visible in the skin as mottling and prolonged capillary refill time and may progress to multiple organ dysfunction syndrome (5).

The skin is the largest organ of the body and may fail under severe physiological stress. Acute skin failure (ASF) denotes the loss of the skin’s barrier and thermoregulatory functions secondary to hemodynamically driven redistribution of blood away from the cutaneous circulation, culminating in necrosis of the skin and subcutaneous tissue (6, 7). ASF develops in 8 to 39% of patients with sepsis (8). Its mechanism is twofold: macrocirculatory collapse reduces peripheral perfusion pressure, and microvascular thrombosis with ischemia–reperfusion injury produces cutaneous hypoxia and necrosis, particularly in distal and pressure-bearing areas (9–11). ASF is associated with prolonged ICU stay, increased treatment costs, and mortality as high as 64% (12), making early detection a clinical priority. A key distinction must be kept explicit: mottling is a clinical sign of cutaneous hypoperfusion that may precede or accompany ASF, whereas ASF denotes failure of barrier function; the two entities are related but not equivalent.

Risk factors for ASF have been studied mainly in mixed critically ill populations (13, 14), and their generalizability to patients with sepsis remains uncertain; sepsis-associated ASF additionally involves gut-skin-brain axis interactions (15). We therefore conducted this systematic review and meta-analysis to determine the incidence of ASF and to identify variables associated with ASF in patients with sepsis, aiming to support early risk identification and prevention.

## 2 Methods

### 2.1 Protocol and registration

This systematic review and meta-analysis was performed following the methodological guidance outlined in the Preferred Reporting Items for Systematic Reviews and Meta-Analysis (PRISMA) statement (16, 17). The protocol was registered in PROSPERO [CRD420251241742].

### 2.2 Literature search

A thorough literature search was conducted across seven electronic databases. Chinese databases comprised the China Knowledge Resource Integrated Database (CNKI), Wanfang Database, and Weipu Database (VIP Database). English databases included the Cochrane Library, PubMed, Web of Science, and Embase. The search covered the period from database inception to December 1, 2025. The 18 included studies were published between 2011 and 2025: 10 within the 5 years preceding the search date, seven between 5 and 10 years, and one (18) more than 10 years earlier; no publication-date restriction was applied, because prospective research on ASF in sepsis is recent, and the publication year of each included study is reported in Table 1. The search strategy was designed to capture studies investigating the incidence rate and risk factors associated with ASF in septic patients. Key search terms were “acute skin failure,” “intensive care units,” “sepsis,” “risk factors,” and “outcome.” The complete search strategies for all seven databases, including the full combinations of MeSH terms and free-text words, are provided in Supplementary Table 1, Appendix 1. Ethics approval was not required as this study utilized solely published data.

**Table 1:** Characteristics of the studies included.

Author/year	Country	Study design	Sample size	Age (years)	Sepsis criteria	ASF criteria	ASF incidence (%)	Factors reported	Mortality (%)	Quality
Ait-Oufella et al. (18)	France	Prospective cohort	60	18–88	①	a	40.0	lactate, urine output, SOFA	45	NOS 7
Ferraris et al. (23)	France	Prospective cohort	46	69 (59–78)	②	a	54.3	lactate, SOFA	30	NOS 6
Ferraris et al. (24)	France	Prospective cohort	43	61 ± 4	①	a	83.7	NE dose, lactate clearance	30	NOS 6
Bi et al. (25)	China	Prospective cohort	172	53.4 ± 14.6	①	a	32.6	temperature, lactate, APACHE II	-	NOS 6
Chen et al. (26)	China	Prospective cohort	162	61 (51–73)	①	b	84.0	APACHE II, SOFA, lactate, CRT > 2 s, ScvO2↓	32.7	NOS 6
Moura et al. (27)	Brazil	Retrospective cohort	97	≥ 18	③	a	39.2	APACHE II, SAPS II, SOFA	45	NOS 5
Dumas et al. (28)	France	Retrospective cohort	259	68 (57–80)	①	a	57.9	lactate, urine output	37	NOS 7
Jing et al. (29)	China	Case–control	123	50 ± 19	④	c	24.4	BSA, lactate	-	NOS 7
Pittman et al. (30)	USA	Case–control	475	57.8 ± 7.7	①	c	41.0	PAD, MV > 72 h, respiratory failure, liver failure	-	NOS 7
Liu et la (9).	China	Retrospective cohort	150	56.6 ± 11.0	①	c	23.3	IL-6, CRP, TNF-α, lactate	-	NOS 7
Jouffroy et al. (31)	France	Retrospective cohort	63	71 ± 14	③	a	30.2	mottling score, CRT, MAP, SOFA	36	NOS 6
Shi et al. (32)	China	Prospective cohort	50	46–89	②	a	36.0	SOFA, APACHE II, lactate	42	NOS 6
Sun et al. (33)	China	Retrospective cohort	154	≥ 18	①	b	31.8	NE dose/duration, ScvO2, oedema	59.2	NOS 7
Tang et al. (34)	China	Retrospective cohort	115	29–98	①	a	30.4	albumin, lactate, MAP	-	NOS 7
Tang et al. (34)*	China	Cross-sectional	254	70.8 ± 15.0	④	b	35.0	PAD, MV, vasoactive drugs, APACHE II, MAP, lactate	33	AHRQ 8
Wang et al. (36)	China	Prospective cohort	491	52.1 ± 9.7	④	b	14.5	BSA, NEWS, SOFA, lactate, PPI, CRT	-	NOS 6
Wang et al. (37)	China	Cross-sectional	123	55.2 ± 21.1	④	b	83.7	BSA, lactate	-	AHRQ 8
Zhang et al. (38)	China	Prospective cohort	458	≥ 18	①	a	48.9	SOFA, APACHE II, lactate, MAP	22.3	NOS 7

Sepsis criteria: ① international consensus definition (Sepsis-3, or the contemporary international consensus for studies published before 2016); ② septic shock (SBP < 90 mmHg or MAP < 65 mmHg despite fluid resuscitation); ③ Surviving Sepsis Campaign criteria; ④ Chinese Guidelines for Emergency Treatment of Sepsis/Septic Shock (2018). ASF criteria: a = mottling score ≥ 3 (mottling-based outcome ascertainment); b = composite clinical criteria (skin mottling, cold clammy skin, CRT > 2 s, decreased transcutaneous oxygen saturation, weakened skin sensation - any one manifestation); c = capillary perfusion pressure < 25 mmHg with characteristic skin plaques. NOS = Newcastle-Ottawa Scale; AHRQ = Agency for Healthcare Research and Quality checklist. * To distinguish another article by the same author (Tang Yan). The two case–control studies (Jing 2023; Pittman 2021) were excluded from the pooled incidence analysis and contributed only to the analysis of factors associated with ASF.

### 2.3 Study selection

Following duplicate removal, two authors (T. L. and K. Z.) independently screened the titles and abstracts of all retrieved records to identify potentially eligible studies. Any record considered relevant by either reviewer underwent a full-text review. The same two authors then independently assessed the full-text articles against the predefined eligibility criteria, and disagreements were resolved through discussion or consultation with a third reviewer (W. S. W.). The eligibility criteria were defined *a priori* as follows. Inclusion criteria: (1) population: adults aged ≥ 18 years with sepsis or septic shock; (2) outcome: ASF ascertained by explicit diagnostic criteria, or mottling score-based ascertainment with a pre-specified threshold, reported as an ASF-related outcome; (3) study design:cross-sectional, cohort, or case–control; (4) reporting:the incidence, associated factors, or clinical outcomes of ASF. Exclusion criteria: (1) duplicate publications or data that could not be extracted; (2) case reports, reviews, and conference papers; (3) publications not in English or Chinese; and (4) studies without full-text availability. Case–control studies fulfilled the inclusion criteria for the analyses of associated factors but were excluded *a priori* from incidence pooling.

### 2.4 Data synthesis and extraction

Two independent reviewers extracted data from the included studies, documenting the following information: first author, publication year, country, study design, sample size, sepsis definition, number of ASF patients, ASF diagnostic criteria, risk factors and outcome data. The sepsis definition applied in each study (Sepsis-3 consensus, Surviving Sepsis Campaign criteria, earlier international consensus definitions, or the Chinese Guidelines for Emergency Treatment of Sepsis/Septic Shock 2018) was recorded and is reported in Table 1. For arterial lactate, effect estimates were harmonized to the odds ratio per 1-mmol/L increase; studies reporting only alternative metrics (e.g., lactate clearance or lactate/albumin ratio) were not entered into the lactate model. All extracted data were stored in Microsoft Excel.

### 2.5 Quality assessment and risk of Bias

Two reviewers (T. L. and K. Z.) independently assessed the methodological quality and risk of bias of the included studies. For cohort and case–control studies, the Newcastle-Ottawa Scale (NOS) was applied, with scores ≥ 7 indicating high quality, 4–6 moderate quality, and < 4 low quality (19). The cross-sectional studies were evaluated using the Agency for Healthcare Research and Quality (AHRQ) checklist (20). A third reviewer (W. S. W.) adjudicated any discrepancies. Inter-rater agreement was high.

### 2.6 Statistical analysis

Statistical heterogeneity was quantified using the *I*^2^ statistic and Cochran’s Q test. *I*^2^ values of 25%, 50%, and 75% were considered to represent low, moderate, and high heterogeneity, respectively. Due to the anticipated clinical and methodological heterogeneity across the included studies, we employed a random-effects model (DerSimonian-Laird) to calculate the pooled incidence and corresponding 95% confidence intervals (CIs).

For the pooled incidence, we additionally reported the 95% prediction interval (PI), which describes the range within which the true incidence of a future study in a similar setting is expected to fall. Subgroup analyses were conducted according to country of origin, study design, and ASF diagnostic criteria (mottling-based outcome ascertainment versus direct ASF diagnostic criteria). A sensitivity analysis, employing a leave-one-out meta-analysis, was performed to evaluate the impact of individual studies on the overall pooled estimate. Publication bias was assessed using Egger’s regression test and Begg’s rank correlation test, and a funnel plot was generated to examine potential asymmetry (21, 22). All analyses were executed using Stata 18.0 software. A two-tailed *p* value < 0.05 was considered statistically significant for all tests.

## 3 Results

### 3.1 Study selection

Our search strategy yielded 3,920 references. Following a thorough assessment of titles, abstracts, and full texts, we identified 18 references that satisfied the inclusion criteria for the meta-analysis, encompassing a total of 3,295 patients (9, 18, 23–38) (Figure 1).

**Figure 1:**
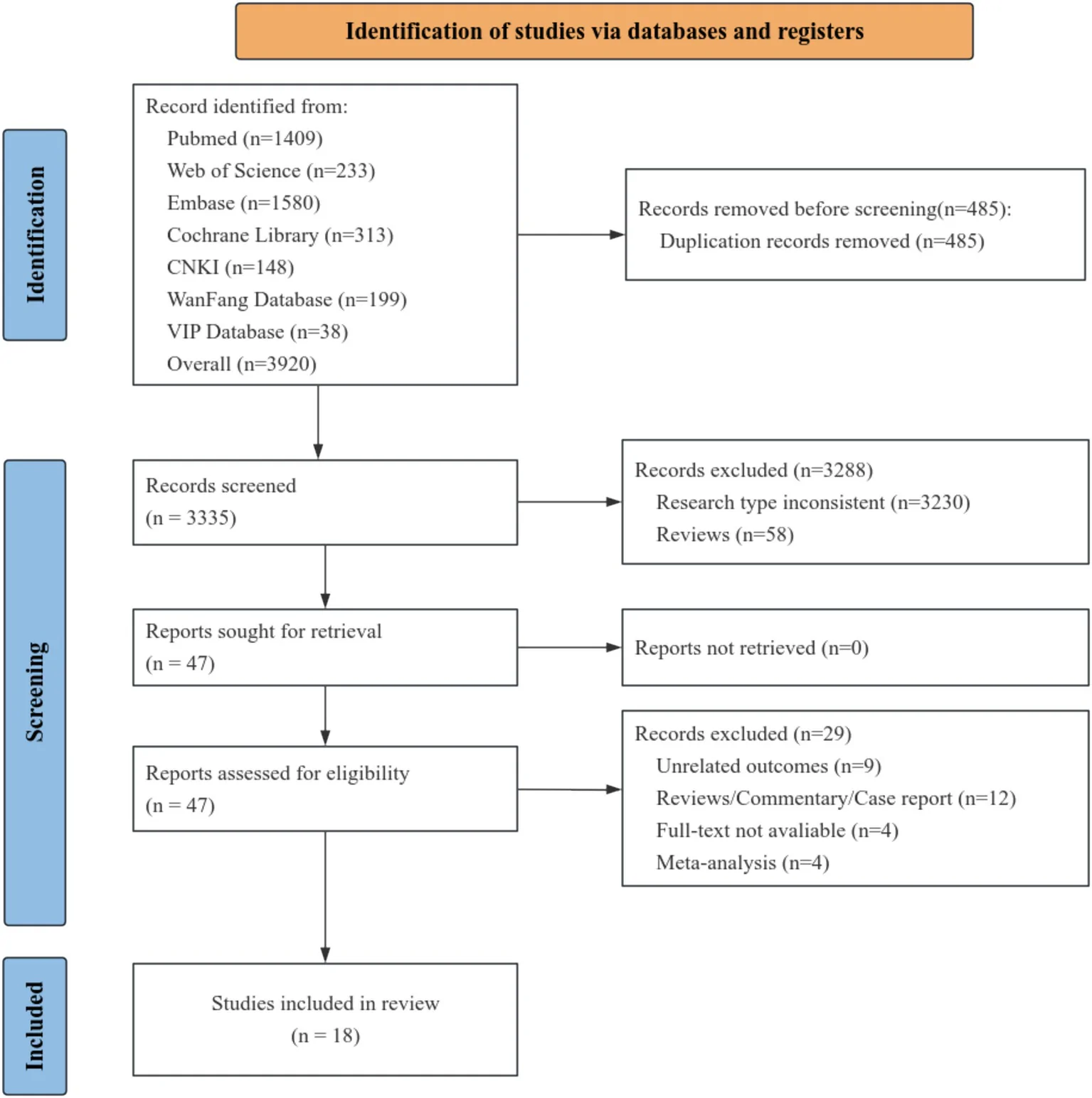
PRISMA flow diagram.

### 3.2 Study characteristics

Among the selected studies, there were eight prospective cohort studies (18, 23–26, 32, 36, 38), six retrospective cohort studies (9, 27, 28, 31, 33, 34), two cross-sectional studies (35, 37), and two case–control studies (29, 30). These studies were conducted between 2011 and 2025 across various countries: 11 in China (9, 25, 26, 29, 32–38), five in France (18, 23, 24, 28, 31), one in the USA (30), and one in Brazil (27). Sepsis was defined according to Sepsis-3 or the contemporary international consensus in 10 studies, by Surviving Sepsis Campaign criteria in two, by clinical septic-shock criteria in two, and by the Chinese Guidelines for Emergency Treatment of Sepsis/Septic Shock (2018) in four (Table 1).

The ASF outcome was ascertained using three types of criteria (Table 1). Ten studies ascertained the outcome using skin mottling scores (mottling score ≥ 3) as a surrogate measure of skin hypoperfusion (18, 23–25, 27, 28, 31, 32, 34, 38); these studies are referred to below as mottling-based outcome studies. Eight studies applied direct ASF diagnostic criteria based on clinical manifestations and pathophysiology (9, 26, 29, 30, 33, 35–37), including composite clinical criteria (skin mottling score, cold clammy skin, capillary refill time > 2 s, decreased transcutaneous oxygen saturation, and weakened skin sensation) or capillary underperfusion pressure < 25 mmHg with characteristic skin plaques; of these eight studies, the two case–control studies (29, 30) were included only in the analysis of associated factors, and the remaining six contributed to the pooled incidence. ASF, mottling and pressure injuries are distinct clinical conditions; the diagnostic criteria used in each study are therefore reported in detail in Table 1, and mottling-based and direct ASF studies were analyzed separately in subgroup analyses.

The distinct attributes of the studies are detailed in Table 1.

### 3.3 Risk of Bias and certainty assessment

The overall quality of the 18 studies varied from moderate to high, with 10 classified as high quality (NOS score ≥ 7 or AHRQ ≥ 8) and 8 as moderate. The results of the quality assessment are presented in Table 1. The level of agreement among authors was high, as indicated by Cohen’s Kappa of 0.88 (95% CI 0.76–0.93). The certainty of the evidence for the outcomes examined was assessed as moderate.

### 3.4 The incidence of ASF

Because incidence cannot be validly estimated from case–control designs, in which sampling is conditioned on outcome status, the two case–control studies (29, 30) were excluded from the pooled incidence analysis and contributed only to the analyses of factors associated with ASF. The main incidence analysis therefore comprised the 16 cohort and cross-sectional studies.

In the 16 cohort and cross-sectional studies included in the main incidence analysis, the incidence of ASF among patients with sepsis ranged from 14.5 to 83.7%. A random-effects meta-analysis estimated the pooled incidence to be 45.2% (95% CI: 34.5–56.2%; *I*^2^ = 96.1%, *p* < 0.0001; Figure 2). The 95% prediction interval was 12.2–83.0%, indicating that the true incidence in a new comparable study could plausibly lie anywhere within this range. Given the very high residual heterogeneity, this pooled estimate should be interpreted with considerable caution and has limited generalizability.

**Figure 2:**
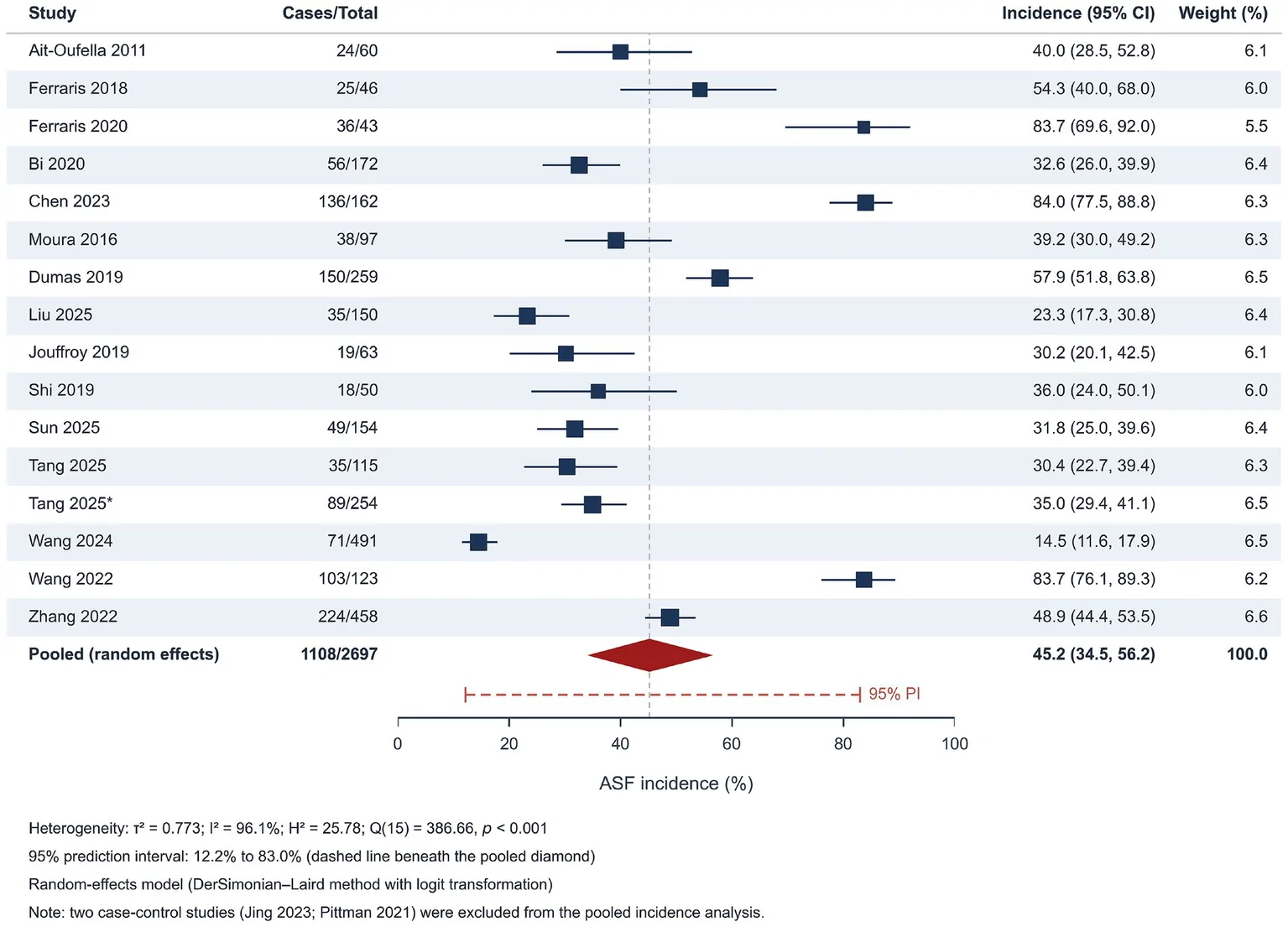
Forest plot of the pooled ASF incidence in patients with sepsis (random-effects model), based on the 16 cohort and cross-sectional studies.

Subgroup analyses were performed according to country of origin, study design, and ASF diagnostic criteria (Table 2). When stratified by outcome ascertainment, the pooled incidence was 44.6% (95% CI: 36.5–53.0%; *I*^2^ = 87.1%) in the ten mottling-based outcome studies and 45.4% (95% CI: 22.8–70.1%; *I*^2^ = 98.3%) in the six studies applying direct ASF diagnostic criteria. By country, the pooled incidence was 41.8% in China, 53.3% in France, and 39.2% in Brazil. By design, the pooled incidence was 49.2% in prospective studies, 35.2% in retrospective studies, and 62.3% in cross-sectional studies.

**Table 2:** Subgroup analyses of the pooled ASF incidence (random-effects model).

Subgroup	*k*	*N*	Pooled incidence (95% CI)	*I* ^2^	95% PI
Main analysis (cohort + cross-sectional)	16	2,697	45.2% (34.5–56.2)	96.1%	12.2–83.0
Mottling-based outcome ascertainment	10	1,363	44.6% (36.5–53.0)	87.1%	22.6–68.9
Direct ASF diagnostic criteria	6	1,334	45.4% (22.8–70.1)	98.3%	5.2–92.6
China	10	2,129	41.8% (28.2–56.7)	97.2%	9.2–83.6
France	5	471	53.3% (38.0–68.0)	87.6%	21.6–82.6
Brazil	1	97	39.2% (30.0–49.2)	-	-
Prospective cohort	8	1,482	49.2% (30.9–67.7)	97.3%	9.1–90.3
Retrospective cohort	6	838	35.2% (24.4–47.7)	91.8%	12.7–67.0
Cross-sectional	2	377	62.3% (15.3–93.8)	98.5%	3.5–98.7

CI, Confidence interval; PI, Prediction interval. The two case–control studies were excluded from all incidence analyses. Subgroup estimates were derived from random-effects models with logit transformation.

### 3.5 Factors associated with ASF

Pooled analyses identified four factors significantly associated with ASF. We identified 27 potential factors reported in at least two studies included in this meta-analysis. Higher arterial blood lactate, higher SOFA and APACHE II scores, and lower MAP were significantly associated with ASF (Figure 3).

**Figure 3:**
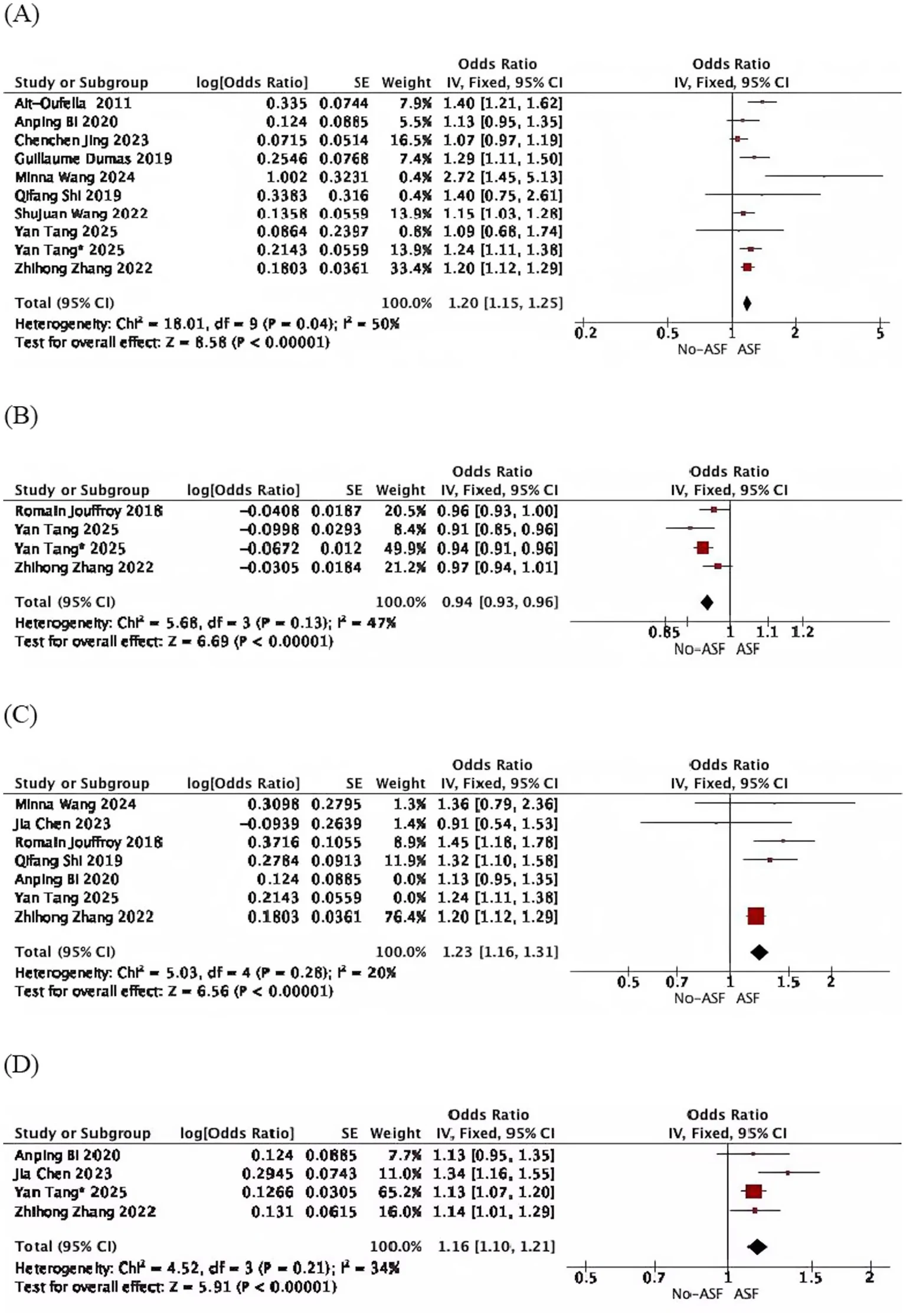
Forest plots of factors associated with ASF: **(A)** arterial blood lactate; **(B)** MAP; **(C)** SOFA score; **(D)** APACHE II score. (A).

Ten studies, comprising a total of 2,105 participants, assessed the association between arterial blood lactate and ASF (18, 25, 28, 29, 32, 34–38). Effect estimates were standardized to the odds ratio per 1-mmol/L increase in arterial lactate; all contributing studies measured arterial rather than venous lactate, although sampling timing (admission versus serial measurements) varied across studies. The pooled estimate indicated that higher arterial blood lactate levels were significantly associated with an increased likelihood of ASF [OR 1.20 (95% CI 1.15–1.25); *p* < 0.00001; *I*^2^ = 50%]. Because *I*^2^ reached the borderline value of 50%, a random-effects model was also fitted as a sensitivity check and yielded a consistent result [OR 1.21 (95% CI 1.13–1.29)].

Four studies, comprising a total of 890 participants, assessed MAP (31, 34, 35, 38). The pooled OR of 0.94 (95% CI: 0.93–0.96; *p* < 0.00001; *I*^2^ = 47%) indicates that lower MAP values were significantly associated with a higher likelihood of ASF.

This meta-analysis also examined the APACHE II score (25, 26, 35, 38) and SOFA score (five studies (25, 26, 31, 32, 36); Figure 3C) to evaluate their association with the development of ASF in sepsis patients. Both the APACHE II and SOFA scores were identified as factors associated with ASF, with ORs of 1.16 (95% CI: 1.10–1.21; *p* < 0.00001; *I*^2^ = 34%) and 1.23 (95% CI: 1.16–1.31; *p* < 0.00001; *I*^2^ = 20%), respectively, indicating that higher scores were associated with increased ASF risk.

### 3.6 Publication Bias and sensitivity analysis

Publication bias was evaluated using Egger’s and Begg’s tests. The results from Egger’s test (*p* = 0.467) and Begg’s test (*p* = 0.239) revealed no evidence of publication bias. In the leave-one-out sensitivity analysis, the pooled incidence estimate ranged from 42.0 to 47.8% when each study was sequentially excluded, indicating that no single study materially altered the pooled estimate.

## 4 Discussion

This systematic review and meta-analysis reveals a high incidence of ASF in sepsis patients, the pooled incidence across the 16 cohort and cross-sectional studies was 45.2% (95% CI: 34.5–56.2%). This finding underscores the significant clinical burden of ASF in septic populations and highlights the critical need for early identification and targeted preventive strategies to mitigate adverse outcomes.

The substantial heterogeneity observed across studies (*I*^2^ = 96.1%) was explored through subgroup analyses based on geographic region, diagnostic criteria, and study design. The pooled incidence was 53.3% in French cohorts, 41.8% in Chinese cohorts, and 39.2% in Brazil. The between-country differences may be attributable to differences in sepsis epidemiology and case mix. Variations in diagnostic approaches were also evident: some studies ascertained the outcome using mottling scores as a surrogate of skin hypoperfusion, whereas others applied composite clinical criteria or perfusion-pressure-based definitions of ASF. Importantly, the pooled incidence was similar between mottling-based outcome studies (44.6%) and direct ASF criteria studies (45.4%), although heterogeneity remained high in both subgroups, and the prediction interval was wide, indicating that the true incidence varies considerably across settings. Regarding study design, cross-sectional studies reported a higher ASF incidence (62.3%) than prospective (49.2%), retrospective (35.2%); prospective protocols with serial standardized skin assessments can capture transient or progressive ASF manifestations often overlooked in single-point evaluations or chart reviews (39).

Our meta-analysis identified four variables significantly associated with ASF development: elevated arterial blood lactate, higher SOFA and APACHE II scores, and lower MAP. These should be interpreted as associations rather than independent or causal risk factors, because most included studies adjusted for different covariate sets and some were unadjusted. Arterial lactate serves as a well-established biomarker for global tissue hypoxia and cellular metabolic dysfunction (40). In sepsis, hyperlactatemia arises not only from microcirculatory failure but also from mitochondrial uncoupling and dysregulated glycolysis, leading to impaired lactate clearance (41). Persistent or rising lactate levels (> 2 mmol/L for > 6 h) signify severe, refractory cellular dysoxia, particularly within the cutaneous microcirculation where perfusion pressure gradients are minimal (42). Thus, serial lactate monitoring is crucial for early risk stratification and timely escalation of hemodynamic support.

MAP represents a potentially modifiable factor: the pooled OR of 0.94 indicates that each 1-mmHg decrease in MAP is associated with a 6% increase in the odds of ASF. Prolonged hypotension, especially MAP < 65 mmHg, triggers compensatory vasoconstriction that diverts blood flow away from the cutaneous microcirculation, leading to tissue hypoperfusion and ischemic damage. Early and sustained MAP optimization is therefore fundamental to resuscitation efforts aimed at preventing ASF.

The SOFA and APACHE II scores, as comprehensive illness severity metrics, reflect the degree of multiorgan dysfunction and systemic inflammation, which collectively compromise cutaneous perfusion and increase ASF susceptibility. Our findings align with previous reports (43) showing that ASF incidence is substantially higher in patients with SOFA scores ≥ 10 compared with lower scores. Combining these scores may enhance risk stratification and facilitate early identification of high-risk patients. While other potential factors, such as body surface area, urine output, and norepinephrine dosage, were identified in individual studies, they lacked sufficient data for pooled analysis, precluding robust effect estimation (44, 45). Their prognostic significance warrants validation in future multicenter studies with standardized ASF definitions and comprehensive covariate adjustment.

This review has several limitations. First, very high heterogeneity (*I*^2^ = 96.1%) was present across studies, attributable to differences in ASF diagnostic criteria, study design, population ethnicity, and sepsis definitions; even after subgroup analyses, heterogeneity remained substantial, and the pooled estimate should be interpreted with caution. Second, the definition of sepsis evolved over the inclusion period, from the earlier international consensus and Surviving Sepsis Campaign criteria to Sepsis-3 and the Chinese Guidelines for Emergency Treatment of Sepsis/Septic Shock (2018); 10 studies applied Sepsis-3-type consensus definitions whereas the remainder used earlier campaign criteria or national guidelines, which affects patient selection and disease severity at enrolment. We addressed this by recording the definition used in each study rather than re-diagnosing patients, which the published data do not permit. Third, the predominance of studies from China and France may limit the generalizability of our findings to other regions. Fourth, due to insufficient data, several potential factors could not be included in the meta-analysis. Fifth, although the lactate model was standardized to the odds ratio per 1-mmol/L increase in arterial lactate, measurement timing (admission versus serial sampling) and assay methods varied across studies. Finally, the observational nature of the included studies precludes causal inference between the identified variables and ASF.

## 5 Conclusion

This meta-analysis demonstrates that the pooled incidence of ASF in sepsis patients is 45.2%, with a wide prediction interval reflecting substantial between-study variation. Arterial blood lactate, SOFA score, APACHE II score and MAP are variables associated with ASF in sepsis patients. These findings provide a preliminary evidence base that may assist healthcare professionals in identifying sepsis patients at heightened risk for ASF. Future prospective studies with standardized ASF definitions are warranted to validate these findings and to develop predictive models for early identification.

## Data Availability

The original contributions presented in the study are included in the article/Supplementary material, further inquiries can be directed to the corresponding author/s.
