# Supplementary material for: Incidence and risk factors of acute skin failure in patients with sepsis: a systematic review and meta-analysis
Source: Front Public Health. 2026 Oct 14;14:1955939. doi: 10.3389/fpubh.2026.1955939 (PMC13713861; doi:10.3389/fpubh.2026.1955939)
Supplement: Supplementary file 1 [file Data_Sheet_1.docx]

**Risk factors and clinical outcomes of acute skin failure in patients with sepsis: A systematic review and meta-Analysis**

**Appendix：Supplementary material**

**Contents**

| **Title** | **Page** |
| --- | --- |
| Appendix 1: Search strategy and databases | 1-3 |
| Appendix 2: Newcastle-Ottawa scale for assessment of quality of included cohort studies. | 4-9 |
| Appendix 3: Publication Bias | 9 |

Appendix 1

Table 1. Search strategy and databases

| Database | Search Terms | No. of studies generated |
| --- | --- | --- |
| Pubmed | ((("Sepsis"[Mesh]) OR (Sepsis Syndrome OR Septicemia OR Septic Syndromes OR Severe Sepsis OR Septic Shock OR Septic Shock, Septic OR Sepsis, Severe OR Blood Poisoning OR Infection, Septic)) AND (("Skin Failure"[Title/Abstract]) OR (Acute Skin Failure OR Skin Dysfunction OR Skin Injury, Acute OR Skin Breakdown OR Skin Integrity Impairment OR Skin Mottling)) AND ((("Risk Factors"[Mesh]) OR (Risk Factor OR Prognostic Factors OR Predictive Factors OR Etiological Factors OR Contributing Factors OR Risk Variables OR Predisposing Factors)) OR (("Clinical Outcome"[Mesh]) OR (Clinical Outcomes OR Patient Outcomes OR Mortality OR Morbidity OR Survival Rate OR Survival OR Length of Hospital Stay OR ICU Stay, Length of OR Hospitalization Duration OR Complications OR Postoperative Complications OR Skin Healing OR Wound Healing OR Quality of Life OR Functional Outcome OR Recovery Outcome))) | 1409 |
| Web of Science | #1 TS=(Sepsis OR Septicemia OR Septicemias OR Sepses OR Septic Syndrome OR Septic Syndromes OR Severe Sepsis OR Severe Septicemias OR Septic Shock)  #2 TS=(Skin Failure OR Skin Failures Failure OR Skin Integrity OR OR Skin Mottling OR Skin Injury OR Kennedy terminal ulcer)  #3 TS=(Epidemiology or Prevalence or Risk Factor or Prognostic Factor or Predictive Factor OR Contributing Factor OR Risk Indicator OR Prognostic Factor OR Risk Determinant)  #4 TS=(Clinical Outcomes OR Patient Outcomes OR Mortalty OR Morbidity OR Survival Rate OR Survival OR Length of Hospital Stay OR ICU Stay Length OR Complication)  #5 #1 AND #2  #6 #5 AND (#3 OR #4) | 233 |
| Embase | ('sepsis'/exp OR 'sepsis' OR 'septic shock'/exp OR 'septic shock' OR 'severe sepsis'/exp OR 'severe sepsis' OR 'septicemia'/exp OR 'septicemia' OR 'kennedy terminal ulcer' OR (kennedy AND terminal AND ('ulcer'/exp OR ulcer))) AND ('skin failure' OR (('skin'/exp OR skin) AND ('failure'/exp OR failure)) OR 'skin injury'/exp OR 'skin injury' OR 'skin integrity'/exp OR 'skin integrity' OR 'skin mottling'/exp OR 'skin mottling') AND ('prevalence'/exp OR 'prevalence' OR 'incidence'/exp OR 'incidence' OR 'risk factor'/exp OR 'risk factor' OR 'prognostic factor'/exp OR 'prognostic factor') AND ('clinical outcome'/exp OR 'clinical outcome' OR 'mortality'/exp OR 'mortality') | 1580 |
| The Cochrane Libary | (((sepsis or septic or septic shock or Septicemia or Severe Sepsis) AND (skin failure or skin mottling or Skin Integrity or Skin Injury or Kennedy terminal ulcer) )AND ( (epidemiology or prevalence or risk factor or Prognostic Factor or Predictive Factor OR Contributing Factor) OR (Clinical Outcomes OR Patient Outcomes OR Mortalty OR Morbidity OR Survival Rate OR Survival OR Length of Hospital Stay OR ICU Stay L length))):ti,ab,kw | 313 |
| CNKI | SU% = ((‘skin failure’ OR ‘end-stage tissue damage’ OR ‘skin dysfunction’ OR ‘skin necrosis’) AND (‘sepsis’ OR ‘septic shock’ OR ‘septicemia’ OR ‘severe sepsis’)) AND ((‘factors’ OR ‘predict’ OR ‘risk’ OR ‘danger’ OR ‘impact’) OR (‘clinical outcome’ OR ‘prognosis’ OR ‘mortality’)) | 148 |
| Wanfang Database | Theme: ((‘skin failure’ OR ‘end-stage tissue damage’ OR ‘skin dysfunction’ OR ‘skin mottling’ OR ‘skin necrosis’) OR (‘sepsis’ OR ‘septic shock’ OR ‘septicemia’ OR ‘severe sepsis’)) AND ((‘factors’ OR ‘predictors’ OR ‘risks’ OR ‘hazards’ OR ‘influences’) OR (‘clinical outcomes’ OR ‘prognosis’ OR ‘mortality’)) | 199 |
| VIP Database | (U = ('skin failure' OR 'end-stage tissue damage' OR 'skin dysfunction' OR 'skin necrosis') AND ('sepsis' OR 'septic shock' OR 'septicemia' OR 'severe sepsis')) AND (U = ('factors' OR 'prediction' OR 'risk' OR 'danger' OR 'impact') OR ('clinical outcome' OR 'prognosis' OR 'mortality')) | 38 |

Appendix 2

**Table 2. Newcastle-Ottawa scale for assessment of quality of included cohort studies.**

| **Study** | **Selection** | | | | **Comparability** | **Outcome** | | | **Total Score** |
| --- | --- | --- | --- | --- | --- | --- | --- | --- | --- |
|  | Representativeness of the Exposed Cohort | Selection of the Non-Exposed Cohort | Ascertainment of Exposure | Demonstration That Outcome of Interest Was Not Present at Start of Study | Comparability of Cohorts on the Basis of the Design or Analysis | Assessment of outcome | Was Follow-Up Long Enough for Outcomes to Occur | Adequacy of Follow Up of Cohorts |  |
| Ait-Oufella,2011 | a | a | a | b | b | a | a | a | 7 |
| Arnaud Ferraris,2018 | a | a | a | b | b | a | a | a | 7 |
| Arnaud Ferraris,2020 | a | a | a | b | b | a | a | a | 7 |
| Bi Anping,2020 | a | a | a | b | b | b | b | d | 6 |
| Chen Jia,2023 | a | a | a | b | a | a | a | a | 8 |
| Edmilson Bastos de Moura,2015 | a | a | a | b | a | a | a | a | 8 |
| Guillaume Dumas,2019 | a | a | a | b | b | a | a | a | 7 |
| Liu Yufei,2025 | a | a | a | b | a | b | b | d | 6 |
| Romain Jouffroy,2018 | a | a | a | b | a | a | a | a | 8 |
| Shi Qifang,2019 | a | a | a | b | b | a | a | a | 7 |
| Sun Meirong,2025 | a | a | a | a | a | a | a | a | 9 |
| Tang Yan,2025 | a | a | a | b | a | a | a | a | 8 |
| Wang Minna,2024 | a | a | a | a | a | b | b | d | 8 |
| Zhang Zhihong,2022 | a | a | a | a | a | b | b | d | 8 |

**Selection**

1) Representativeness of the exposed cohort

a) truly representative of the average	(describe) in the community *

b) somewhat representative of the average	in the community *

c) selected group of users eg nurses, volunteers

d) no description of the derivation of the cohort

2) Selection of the nonexposed cohort

a) drawn from the same community as the exposed cohort *

b) drawn from a different source

c) no description of the derivation of the non exposed cohort

3) Ascertainment of exposure

a) secure record (eg surgical records) *

b) structured interview *

c) written self report

d) no description

4) Demonstration that outcome of interest was not present at start of study

a) yes *

b) no

**Comparability**

1) Comparability of cohorts on the basis of the design or analysis

a) study controls for	(select the most important factor) *

b) study controls for any additional factor * (This criteria could be modified to indicate specific control for a second important factor.)

**Outcome**

1) Assessment of outcome

a) independent blind assessment *

b) record linkage *

c) self report

d) no description

2) Was follow-up long enough for outcomes to occur

a) yes (select an adequate follow up period for outcome of interest) *

b) no

3) Adequacy of follow up of cohorts

a) complete follow up - all subjects accounted for *

b) subjects lost to follow up unlikely to introduce bias - small number lost - > % (select an adequate %) follow up, or description provided of those lost) *

c) follow up rate <	% (select an adequate %) and no description of those lost

d) no statement

**Table 3. Newcastle-Ottawa scale for assessment of quality of included case-control studies.**

| **Study** | **Selection** | | | | **Comparability** | **Outcome** | | | **Total Score** |
| --- | --- | --- | --- | --- | --- | --- | --- | --- | --- |
|  | Is the case definition adequate? | Representativeness of the cases. | Selection  of Controls. | Definition of Controls. | Comparability of cases and controls on the basis of the design or analysis. | Ascertainment of exposure | Same method of ascertainment for cases and controls | Non-Response rate |  |

| Jing Chenchen，2023 | a | a | a | a | a | a | a | a | 8 |
| --- | --- | --- | --- | --- | --- | --- | --- | --- | --- |
| Joyce Pittman，2021 | a | a | a | a | a | a | a | a | 8 |

**Selection**

1) Is the case definition adequate?

a) yes, with independent validation *

b) yes, eg record linkage or based on self reports

c) no description

2) Representativeness of the cases

a) consecutive or obviously representative series of cases *

b) potential for selection biases or not stated

3) Selection of Controls

a) community controls *

b) hospital controls

c) no description

4) Definition of Controls

a) no history of disease (endpoint) *

b) no description of source

**Comparability**

1) Comparability of cases and controls on the basis of the design or analysis

a) study controls for	(Select the most important factor.)*

b) study controls for any additional factor * (This criteria could be modified to indicate specific control for a second important factor.)

**Exposure**

1) Ascertainment of exposure

a) secure record (eg surgical records) *

b) structured interview where blind to case/control status *

c) interview not blinded to case/control status

d) written self report or medical record only

e) no description

2) Same method of ascertainment for cases and controls

a) yes *

b) no

3) Non-Response rate

a) same rate for both groups *

b) non respondents described

c) rate different and no designation

Appendix 3

Publication Bias

Fig.1. The Funnel Chart
